# An integrated and continuous downstream process for microbial virus‐like particle vaccine biomanufacture

**DOI:** 10.1002/bit.28118

**Published:** 2022-05-10

**Authors:** Lukas Gerstweiler, Jagan Billakanti, Jingxiu Bi, Anton P. J. Middelberg

**Affiliations:** ^1^ School of Chemical Engineering and Advanced Materials The University of Adelaide Adelaide South Australia Australia; ^2^ Global Life Sciences Solutions Australia Pty Ltd. Parramatta New South Wales Australia; ^3^ Division of Research and Innovation The University of Adelaide Adelaide South Australia Australia

**Keywords:** biomanufacture, cont, continuous downstream processing, process integration, vaccine, virus‐likeparticle

## Abstract

In this study, we present the first integrated and continuous downstream process for the production of microbial virus‐like particle vaccines. Modular murine polyomavirus major capsid VP1 with integrated J8 antigen was used as a model virus‐like particle vaccine. The integrated continuous downstream process starts with crude cell lysate and consists of a flow‐through chromatography step followed by periodic counter‐current chromatography (PCC) (bind‐elute) using salt‐tolerant mixed‐mode resin and subsequent in‐line assembly. The automated process showed a robust behavior over different inlet feed concentrations ranging from 1.0 to 3.2 mg ml^−1^ with only minimal adjustments needed, and produced continuously high‐quality virus‐like particles, free of nucleic acids, with constant purity over extended periods of time. The average size remained constant between 44.8 ± 2.3 and 47.2 ± 2.9 nm comparable to literature. The process had an overall product recovery of 88.6% and a process productivity up to 2.56 mg h^−1^ ml_resin_^−1^ in the PCC step, depending on the inlet concentration. Integrating a flow through step with a subsequent PCC step allowed streamlined processing, showing a possible continuous pathway for a wide range of products of interest.

## Graphical abstract

In this work an integrated and continuous downstream process for microbially expressed virus‐like particle vaccines was developed, combining a flow through with a periodic counter‐current chromatography and in‐line assembly. The decribed process produces virus‐like particles of constant high quality and shows a possible pathway for continuous biomanufacturing of therapeutic proteins.

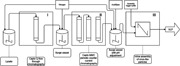

## 1 INTRODUCTION

Viral structural proteins can self‐assemble into particles that correspond to the overall appearance of native viruses, yet lacking in genetic material. These so‐called virus‐like particles (VLPs) are therefore unable to replicate and thus are considered nonpathogenic (Donaldson et al., 2018). Due to their native capsid structure, VLPs can induce strong humoral and cellular immune responses without the need for adjuvants, making them powerful candidates for future vaccines (Pattenden et al., 2005; Rivera‐Hernandez et al., 2013; Stanley, 2006). Another key benefit of VLPs as vaccine candidates is the possibility to insert foreign antigens to construct vaccine candidates against all types of diseases while the underlying VLP construct remains the same. VLPs are therefore extensively examined as vaccine candidates against pathogens such as Influenza, Rotavirus, Group A Streptococcus, and others, and also are commercial products against human papillomavirus, malaria, and hepatitis B/E (Anggraeni et al., 2013; Laurens, 2020; Nooraei et al., 2021; Rivera‐Hernandez et al., 2013). A disadvantage, however, is the rather high costs of VLPs caused by complicated production and purification processes (Effio & Hubbuch, 2015; Hume et al., 2019; Qendri et al., 2019). Downstream processing is often based on size exclusion chromatography and ultra‐centrifugation that might face challenges in large‐scale set‐ups (Effio & Hubbuch, 2015; Tayyab et al., 1991). Another challenge is low binding capacities during chromatography in bind‐and‐elute mode (Effio & Hubbuch, 2015).

To overcome some of the production challenges and to intensify the production of VLP vaccines, platform technologies have been developed that allow the production of a variety of VLPs while only requiring minimal adjustments of the underlying production process (Middelberg et al., 2011). One possible pathway is to produce VLPs and antigens separately and subsequently attach them either by conjugation or by tag coupling approaches (Brune et al., 2016). Another pathway is genetic fusion in which the antigen is genetically inserted into the viral structural protein with subsequent protein expression as one construct (Clarke et al., 1987; Sapsford et al., 2013). An advanced platform technology for VLP vaccines involves the use of murine poliomavirus major capsid protein VP1 with inserted antigen (Middelberg et al., 2011). VLP vaccines based on this platform showed promising results in animal studies for pathogens such as Influenza, Group A Streptococcus, and Rotavirus (Anggraeni et al., 2013; Seth et al., 2016; Tekewe et al., 2017). The capsomeres can be expressed unassembled in gram‐per‐liter concentration in *Escherichia coli* and a highly efficient, scalable, and integrated purification and production pathways have been developed (Gerstweiler et al., 2021c; Liew et al., 2010).

Continuous bio‐processing promises process intensification due to higher automation, increased equipment utilization and a reduced facility footprint, and furthermore leads to constant product quality and less batch‐to‐batch variation. A review on current developments of continuous bio‐manufacturing has been recently published (Gerstweiler et al., 2021a). Despite its promises, continuous processing is not widely utilized within bio‐pharmaceutical processing yet, but gains more and more attention within scientific and industrial communities. Most research focuses on the transition of existing batch unit operations to continuous ones. Strategies such as periodic counter‐current chromatography (PCC), multi‐column solvent gradient purification (MCSGP) and simulated moving bed (SMB) for chromatography, counter‐current mixer settlers for extraction, coiled‐flow inverter and tubular reactors for precipitation, and single‐pass tangential flow filtration for filtration have been developed and described in the literature (Arunkumar et al., 2018; Burgstaller et al., 2019; Godawat et al., 2012; Kateja et al., 2016; Kröber et al., 2013; Martínez Cristancho & Seidel‐Morgenstern, 2016; Rosa et al., 2013; Sun et al., 2020). Integrated continuous processes, however, are seldom described and usually focus on antibody purification. For example, Godawat et al. (2015) coupled two PCC (protein A and CEX) steps with a flow‐through step, while Steinebach et al. (2017) integrated a PCC process with MCSGP and a flow‐through polishing step to purify antibodies. As far as we are aware, an integrated continuous production pathway for VLPs formed by self‐assembly ex vivo has not been developed or described.

To further extend the field of continuous bio‐processing we developed and here report an automated continuous and integrated purification process for microbially‐expressed VLP vaccines based on an integrated production pathway developed by our group (Gerstweiler et al., 2021c). The process couples a flow through Capto™ Q chromatography step followed by a bind‐elute multimodal (Capto™ MMC) PCC process with subsequent in‐line assembly of VLPs. It has been previously shown that nonpurified VP1 capsomeres form soluble aggregates with microbial DNA at low buffer salt concentrations, hindering purification (Gerstweiler et al., 2021b). In batch processing, the use of salt‐tolerant mixed‐mode resins with a previous flow‐through step allows processing at elevated salt concentrations, which suppress VP1‐DNA aggregation, and therefore leads to better recovery. The salt‐tolerant mixed‐mode resin furthermore allows an integration of the two‐unit operations without buffer adjustment in between and enables a wide design space (Gerstweiler et al., 2021b, 2021c). The continuous process described in this study, developed by building on these batch studies, produces VLPs of a constant good quality, removes DNA and most contaminants, is scalable and can act as a platform technology for the development for new continuous production pathways of vaccines and biopharmaceuticals other than monoclonal antibodies.

## 2 MATERIALS AND METHODS

### 2.1 Protein expression and sample preparation

Murine polyomavirus major capsid protein VP1 with inserted Group A Streptococcus antigen J8 was constructed and expressed as described in our earlier paper (Gerstweiler et al., 2021c). In brief, GCN4‐J8 was inserted with flanking G4S linkers into VP1 and cloned into pETDuet‐1 and transformed by heat shock transformation into Rosetta™ 2(DE3) Singles™ competent cells (Merck KGaA) and stored as 25% glycerol stocks. Cell stock was grown overnight in Terrific Broth (TB) medium (12 g l^−1^ tryptone (LP0042, Thermo Fisher Scientific), 24 g l^−1^ yeast extract (P0021, Thermo Fisher Scientific), 5 g l^−1^ Glycerol (GL010, ChemSupply), 2.31 g l^−1^ potassium dihydrogen phosphate (PO02600, ChemSupply) and 12.5 g l^−1^ dipotassium hydrogen phosphate (PA020, ChemSupply) with 35 µg ml^−1^ chloramphenicol (GA0258, ChemSupply) and 100 µg ml^−1^ ampicillin (GA0283, ChemSupply) at 37°C and 200 rpm, 1 l shake flask, 200 ml medium. Next morning overnight culture was diluted 1:40 into fresh TB media and grown in 1 l shake flasks each containing 200 ml of fermentation broth at 37°C and 200 rpm. After reaching an optical density OD_600_ of 0.5, the temperature was reduced to 27°C and protein expression was induced by IPTG (15529019, Thermo Fisher Scientific) addition to a final concentration of 0.1 mM After 16 h expression, the cells were harvested by centrifugation, cell paste was washed once with 0.9% w w^−1^ sodium chloride (SL046, ChemSupply) and stored at −80°C in 50 ml aliquots until subsequent use.

To obtain clarified cell lysate, cells were resuspended in lysis buffer (20 mM Tris‐hydrochloride (GB4431, ChemSupply), 1 mM EDTA (EA023, ChemSupply), 5% w w^−1^ glycerol, 5 mM dithiothreitol (DTT) (DL131, ChemSupply), pH 8.9) with 1x SigmaFast™ protease inhibitor (SA8820 Millipore Sigma), lysed by ultrasonication (Scientz‐IID), centrifuged twice for 30 min at 20,130 g, 4°C (A5920R centrifuge, Eppendorf) and filtered (0.45 µm, Minisart®, Sartorius). After filtration NaCl was added to a final concentration of 0.35 M and diluted with lysis buffer pH 8.9 containing 0.35 M NaCl as needed. Clarified lysate was stored on ice during processing.

### 2.2 In process analytics

Total protein concentration was measured using the Bradford assay at 595 nm (BioRad Laboratories) in 96‐well plates (200 µl) with bovine serum albumin (BSA) as a standard. BSA standard concentration was verified by A_280_ absorbance on a NanoDrop™ (Thermo Fisher Scientific).

Host cell DNA was measured with the Quant‐iT™ broad range dsDNA Assay Kit (Q33130, Thermo Fisher Scientific), with fluorescence (485/530 nm) measured on a 2300 Victor X5 multilabel reader (PerkinElmer).

RP‐HPLC was used to determine VP1‐J8 concentration as previously described (Gerstweiler et al., 2021c). In brief samples were combined 1:4 with a denaturing buffer (8 M guanidine [GE1914, ChemSupply], 50 mM DTT, 20 mM Tris pH 8) and heated for 10 min at 75°C. A gradient elution with water, containing 0.5% TFA (Buffer A) (TS181, ChemSupply), and acetonitrile (LC1005, ChemSupply), containing 0.4% TFA (Buffer B), was used to separate the sample (3 µl) on a Vydac Protein C4 column 2.1 × 100 mm, 5 µm (214TP521), at a flow of 1 ml min^−1^ and 60°C column temperature. The elution program was as following: 6 min gradient from 35% B to 60% B, 30 s gradient from 60% B to 100% B, 1 min 100% B, 30 s from 100% B to 35% B, and 4 min of 35% B (Gerstweiler et al., 2021c). A Shimadzu UFLC‐XR system (pump: LC‐20AD‐XR, autosampler: SIL‐20AXR, diode array detector: SPD‐M20A, column oven: CTO‐20) with detection at 280 nm was used for HPLC experiments.

Size and shape of VLPs were examined under transmission electron microscopy (TEM). Samples (10 µl) were diluted 1:10 with MilliQ water. A drop of 5 µl was put on plasma cleaned carbon‐coated square meshed grids (GSCU100C, ProSciTec) and incubated at room temperature for 5 min. The sample was removed with blotting paper and the grid was washed twice with water before stained with 2% w v^−1^ uranyl acetate for 2 min. Images were taken with a FEI Tecnai G2 Spirit with an Olympus SIS Veleta CCD camera at 120 kV voltage. Sizes were measured by counting pixels with GIMP 2.10.18. The pixel size has been calibrated with a carbon‐grated replica grid.

Reducing sodium dodecyl sulfate–polyacrylamide gel electrophoresis (SDS‐PAGE) was conducted with TruPAGE™ precast Gels 12%, 10 × 8 cm 12‐well (PCG2010, Millipore Sigma) following the manufacturer's recommendations. Equal volumes of samples (6 µl) were used for all runs with Precision Plus Protein™ Standard (1610363, Bio‐Rad) as a size standard.

Dynamic light scattering (DLS) was conducted on a Zetasizer NanoZS (Malvern Panalytical). Samples of 500 µl, equilibrated at 20°C for 5 min were measured. Each measurement is the average of 100 measurements of one sample. The refractive index of the dispersant was assumed to be 1.33 with a viscosity of 1.02 cp (Chuan et al., 2008). As a fitting algorithm nonnegative constrained least squares (NNLS) fitting algorithm was used.

### 2.3 Flow‐through chromatography

Continuous flow‐through chromatography was conducted in repeating cycles of flow‐through chromatography starting with clarified cell lysate of different total protein concentrations as described on a 5 ml prepacked HiTrap™ Capto™ Q column (Cytiva) on an AKTA™ AVANT™ system (Cytiva). One cycle consisted of the following steps: 1.2 CV equilibration (lysis buffer pH 8.9 containing 0.35 M NaCl) at 5 ml min^−1^, followed by sample loading til 90% of DBC_1%_ at 2 ml min^−1^ and a subsequent postloading wash with equilibration buffer (1 CV). This was followed by a wash cycle of 2 CV H_2_O (10 ml min^−1^), 17 CV 1 M NaOH in reverse flow (5 ml min^−1^), 2 CV of H_2_O (10 ml min^−1^), and 5 CV equilibration (5 ml min^−1^). The maximum loading volume was dependent on the clarified lysate concentration determined during the first cycle. The maximum loading volume was set as a loading volume for subsequent processing. To minimize dilution the flowthrough peak was collected as shown in Appendix A1. Recovery was determined in a separate experiment for 10 cycles with clarified lysate having a total protein concentration of 3 mg ml^−1^ as a starting material, as this was the highest concentration used in the experiments.

### 2.4 Periodic counter‐current chromatography

Continuous periodic counter‐current chromatography in a 3C‐PCC setup was performed on an AKTA™ PCC™ system (Cytiva, Sweden) with 1 ml prepacked HiTrap™ Capto™ MMC columns. The collected flowthrough from the previous Capto™ Q chromatography run, which was collected in a stirred vessel (50 ml bottle on ice, 50 rpm, 20 mm stirring bar), was used without any further adjustment for bind‐elute processing. To determine the design space, breakthrough curves at flowrates of 1 ml min^−1^ (1 min contact time), 0.5 ml min^−1^ (2 min contact time), and 0.25 ml min^−1^ (4 min contact time) were measured at a VP1‐J8 concentration of 0.71 mg ml^−1^ and the maximum possible overloading calculated, according to the area under the breakthrough curve method (Godawat et al., 2012; Löfgren et al., 2021). The maximum overloading can be defined as being when the integral of the breakthrough curve equals the dynamic binding capacity. For the actual continuous processing, the switching times of the PCC process were controlled based on the dynamic UV control method developed by Cytiva (Bangtsson & Lacki, 2012) using column inlet UV absorbance and the column outlet UV absorbance of the first column in a connected set‐up to calculate the column breakthrough. However, we found that the proposed method with ΔUV as a controlling signal is error prone if the inlet feed concentration is not constant, which is a result of the integrated process; therefore, loading to approximately 70% breakthrough was controlled with a new approach developed by our group (Gerstweiler et al., 2022). A cycle of the PCC process consists of the following phases: Loading at a flowrate between 0.4 and 0.8 ml min^−1^ til breakthrough level triggered; Post loading wash of 2 CV at 1 ml min^−1^; Washing with 5 CV at 1 ml min^−1^ with equilibration buffer containing no DTT (lysis buffer pH 8.9 containing 0.35 M NaCl); Elution with 20 CV of elution buffer (40 mM di sodium hydrogen phosphate (SA026, ChemSupply), 1 M NaCl, 5% w w^−1^ glycerol, 1 mM EDTA) at 1 ml min^−1^; Cleaning with 1 M sodium hydroxide (SA178, ChemSupply) for 15 CV at 1 ml min^−1^; Washing with water for 5 CV at 1 ml min^−1^, and; Re‐equilibration for with equilibration buffer for 5 CV at 1 ml min^−1^. Recovery was measured in a separate experiment with a loading material containing 0.32 mg ml^−1^ VP1‐J8 at a flow rate of 0.45 ml min^−1^.

### 2.5 Assembly of VLPs

The elution of the PCC process was collected in a 50 ml stirred vessel (20 mm stirring bar, 50 rpm). The vessel was prefilled with approximately 10 ml of elution buffer, containing no product, to submerge the sensor. As described in Section 2.4, the elution buffer does not contain stabilizing DTT, which supresses VLP assembly. This approach to change the buffer system during elution allowed the removal of DTT without a dedicated buffer exchange step (Gerstweiler et al., 2021c). The pH value in the vessel was controlled at a value of pH 7.2. This was implemented with a BioFlo® 320 control panel (Eppendorf, Germany) with 1% v v^−1^ HCl, 1 M NaCl, 5% w w^−1^ glycerol as an acid solution and 0.2 M NaOH, 1 M NaCl, 5% w w^−1^ glycerol as a base solution. The pH adjusted VP1‐J8 solution was continuously used as inlet A on an AKTA^TM^ Go system (Cytiva) and mixed 9:1 in‐line with assembly trigger solution (30 mM calcium chloride (CA033, ChemSupply), 1 M NaCl, 5% w w^−1^ glycerol). After dilution and neutralizing remaining EDTA this achieves a final calcium ion concentration of 2 mM. The outlet was fractionated every 5 ml and incubated for 24 h before storage at −80°C.

### 2.6 Process integration and experimental set‐up

**Figure 1:**
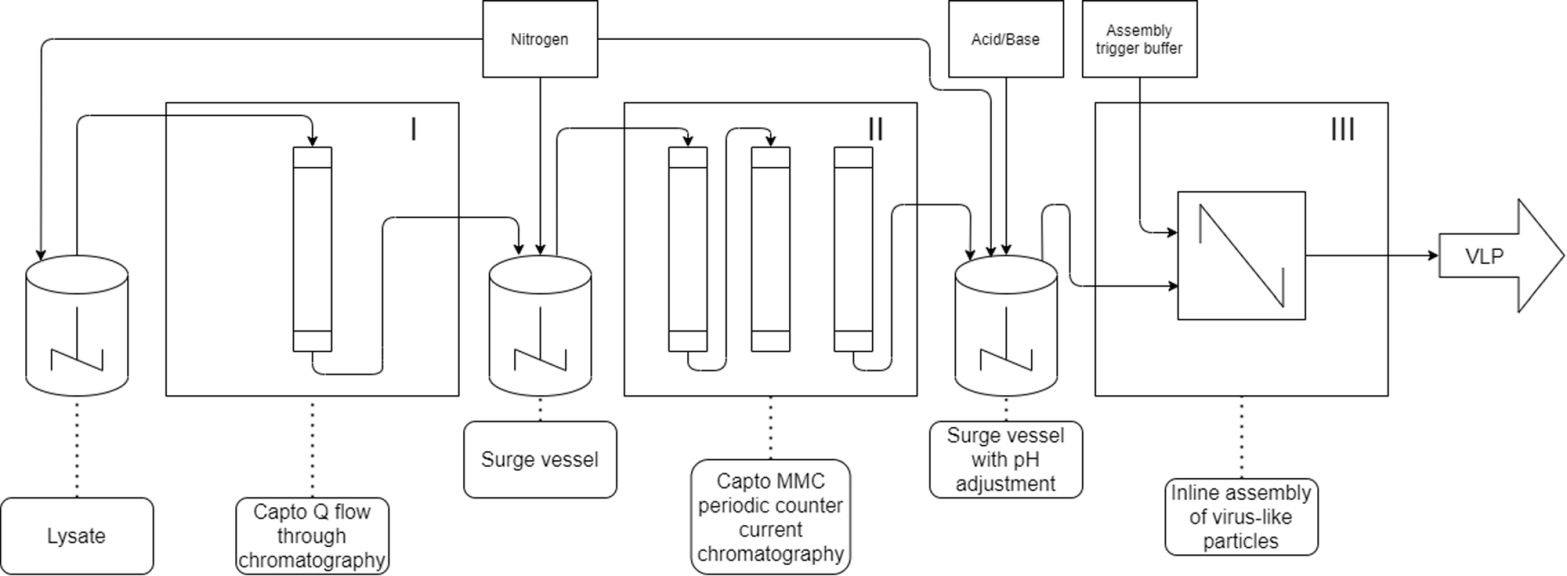
Flow chart of continuous process for the production of microbial virus‐like particles (VLPs) consisting of three integrated unit operations. The clarified lysate containing viral capsomeres is first purified on Capto™ Q in flow‐through mode (I, ÄKTA™ AVANT™) and collected in a surge vessel. The collected flow through is then continuously loaded on Capto™ MMC columns in a continuous periodic counter‐current chromatography process (II, ÄKTA™ PCC™). The elution containing purified capsomeres is collected in a surge vessel and pH adjusted, after which it is mixed in‐line with assembly trigger buffer (III, ÄKTA™ GO™) to initiate assembly of VLPs. Preassembly the solutions are fumigated with nitrogen to prevent oxidation.

Figure 1 shows an overall flow chart of the VLP purification process; a picture of the whole set‐up can be found in Appendix A2. The three‐unit operations used in this workflow—Capto™ Q in flow‐through chromatography, Capto™ MMC PCC in bind‐elute chromatography, and in‐line assembly of VLPs—were coupled with surge vessels (50 ml glass bottles with 20 mm magnetic stirring bar, [Schott AG]) at 50 rpm. The overall footprint of the whole downstream processing set‐up was approximately 4 m of laboratory bench space for up to 7.56 mg h^−1^ overall productivity (2.52 mg h^−1^ ml_resin_^−1^ at the PCC step). A 5 ml HiTrap Capto™ Q and 3 ×1 ml Capto™ MMC columns were used. The surge vessels as well as the clarified cell lysate were constantly flushed with nitrogen to prevent undesired protein oxidation. To show the robustness of the process, experiments with three different initial total protein concentrations of clarified cell lysate (1.0, 2.2, and 3.2 mg ml^−1^) were conducted. The only process parameters that were changed were flow rates of the PCC process and assembly process to match the output of the flow‐through step. Each experiment was run between 10 and 12 h. The process parameters are summarized in Table 1.

**Table 1:** Process parameter of conducted experiments.

	Experiment 1	Experiment 2	Experiment 3
Total protein concentration (mg ml^−1^)	1.0	2.2	3.2
DNA concentration (µg ml^−1^)	54.4	94.2	154.0
VP1 concentration (mg ml^−1^)	0.14	0.32	0.41
Loading flow through per cycle (ml)	25	17	14
PCC loading flow rate (ml min^−1^)	0.7	0.5	0.45
VLP Assembly flow rate (ml min^−1^)	0.22	0.2	0.2

Abbreviations: PCC, periodic counter‐current chromatography; VLP, virus‐like particles.

## 3 RESULTS AND DISCUSSION

### 3.1 Breakthrough experiments and PCC design

**Figure 2:**
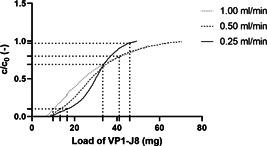
Breakthrough curves for VP1‐J8 loading onto a 1 ml prepacked Capto™ MMC column at different flow rates. Maximum overloading for periodic counter‐current chromatography processes based on the area under the curve method are highlighted with dotted lines, as well as DBC_10%_ as a reference for batch production.

Breakthrough experiments performed to determine process design space of the PCC process are illustrated in Figure 2. The breakthrough curve obtained for a flow rate of 0.25 ml min^−1^ (4 min residence time) demonstrated as expected a sigmoidal shape. However, in contrast, the breakthrough curve at 1 ml min^−1^ resembled more a logarithmic function. The curve for 0.5 ml min^−1^ describes a shape between the two aforementioned curves. This behavior can be explained by the large size of VP1‐J8 of 232 kDa and the resulting mass transfer limitations. This effect has been well described for other large biomolecules elsewhere in the literature (Hahn et al., 2005; Ng et al., 2012; Swinnen et al., 2007). The DBC_10%_ values decreased from 16.6 mg ml^−1^ to 13.3 mg ml^−1^ to 9.9 mg ml^−1^ with increasing flow rates of 0.25, 0.5, and 1.0 ml min^−1^, respectively. Similarly, the maximum column breakthrough until the column became overloaded in a PCC process decreased from 98% to 80% to 69%. The chosen trigger breakthrough level of 70% for column switching ensured robust processing without significant product loss for the investigated flow rates between 0.25 and 1 ml min^−1^. Lower flow rates would result in unreasonably long cycle times while higher flow rates bear the risk of product loss in the flow through. Recently it was shown that optimal process conditions in continuous twin column processes can be found at relatively low column residence times of between 1 and 2 min and column breakthroughs between 50% and 80% for low and medium product concentrations (<5 mg ml^−1^). This is, however, highly dependent on the type of resin used and the desired product concentration in the feed stream, and it is thus hard to generalize (Sun et al., 2021).

### 3.2 Evaluating integrated process performance

During the flowthrough chromatography step, a total of 60.3 ± 1.8 mg VP1 was applied to the Capto™ Q column and 60.0 ± 1.2 mg could be recovered, resulting in a recovery of 99.5% for this step. This high recovery is expected as the product is not binding to the resin and thus losses are expected to be minimal. For the PCC step in bind‐elute mode, out of 31.0 ± 0.62 mg VP1 applied on Capto™ MMC, 27.6 ± 1.38 mg (89.0%) was recovered in the elution pool and approximately 0.73 ± 0.12 mg (2.4%) remained in the flow through. The product loss in the flow‐through probably corresponds to aggregated VP1‐J8 capsomeres, as we have recently shown that VP1‐J8 aggregates do not bind to Capto™ MMC and remain in the flow‐through (Gerstweiler et al., 2021c). The remaining 8.4% is either strongly bound to the matrix or noneluted with selected conditions or stripped in the washing step with 1 M NaOH. A longer elution time might help to further increase the recovery at the cost of a lower product concentration. Assembly into VLPs is triggered solely by the addition of assembly trigger buffer and therefore a 100% recovery for the assembly step is assumed. This results in an overall process recovery of 88.6%, with a high product purity and quality as described in detail in Section 3.3. This is comparable to another process for HBCaAg VLPS achieving 86% recovery (Hillebrandt et al., 2020) and is significantly higher than other described batch processes for the production of VLPs that reported recoveries between 31% and 76% (Carvalho et al., 2019; Hillebrandt et al., 2020; Ladd Effio et al., 2015; Zhao et al., 2015).

**Figure 3:**
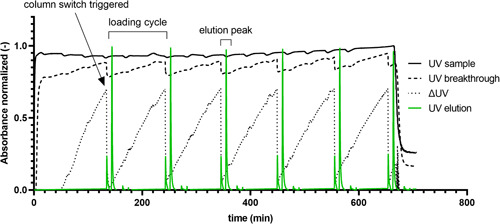
Chromatogram of continuous periodic counter‐current chromatography in bind and elute mode integrated with a prior flow‐through step. Starting material had an initial total protein concentration of 3.2 mg ml^−1^. Shown are the UV sample signal (output of flow‐through chromatography), UV breakthrough profile of the overloaded column, the calculated column product breakthrough (ΔUV), and UV elution step signals. The smaller peak previous of the elution peak is a signal caused by the postloading wash step, and is not eluted but collected on the second column. Values normalized to 1 (UV sample and UV breakthrough to maximum UV sample value, UV elution to maximum UV elution, ΔUV divided by 100).

A chromatogram from the 3‐column PCC unit operation of the third experiment having an initial total protein concentration of 3.2 mg ml^−1^ is shown in Figure 3. As can be seen the concentration of the inlet stream obtained from the surge vessel after Capto™ Q flow‐through chromatography fluctuates slightly, caused by the cyclic nature of the prior flow‐through unit operation. The control strategy developed for unsteady inlet feed concentrations, reliably triggered column switching (Gerstweiler et al., 2022).

**Figure 4:**
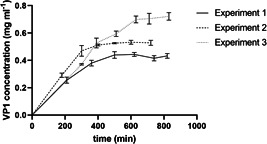
VP1‐J8 concentration in surge vessel after periodic counter‐current chromatography processing but before assembly. Runs were conducted with initial protein concentrations of 1 mg ml^−1^ (Experiment 1), 2.2 mg ml^−1^ (Experiment 2), and 3.2 mg ml^−1^ (Experiment 3).

Figure 4 shows the VP1‐8 concentration in the surge vessel after PCC processing, in which the pH value is adjusted (see Figure 1). The VP1‐J8 concentration increases in the first 400–600 min before it reaches a stable level. This equilibration time is a result of prefilling the surge vessel with buffer, containing no product, to submerge the pH sensor. Although column switching in the PCC system is triggered at 70% product breakthrough and at a constant elution volume in all experiments, the VP1‐J8 concentration equilibrates at different values in each experiment. As can be seen in Figure 4 the VP1‐J8 concentration in the surge vessel equilibrates at concentrations of 0.43, 0.53, and 0.70 mg ml^−1^ for initial total protein concentrations of the lysate of 1.0, 2.2, and 3.2 respectively. Thus, a higher product concentration in the inlet feed stream also translates to a higher product concentration in outlet stream if elution volume and column breakthrough remain constant. This can be explained by a nonconstant binding capacity in the concentration range used in these experiments. The VP1‐J8 concentration in the inlet feed of the PCC was only between 0.12 mg ml^−1^ (Experiment 1) and 0.37 mg ml^−1^ (Experiment 3) and assuming a Langmuir‐like binding behavior literature suggests a high dependence of the product concentration on the binding capacity in low concentration ranges (Latour, 2015; Yu et al., 2014). If a constant product concentration in the surge vessel is desired, the elution volume could be controlled. Namely, the elution volume could be decreased at low product concentrations or increased at higher product concentrations to obtain the same average product concentration.

Interestingly an increase in initial feed stream concentration does not translate linear increase in the overall productivity of the process. Doubling the initial protein concentration from 1 to 2.2 mg ml^−1^ (Experiments 1 and 2) only increased the overall productivity after assembly by 12%, from 5.1 to 5.7 mg h^−1^ (1.7, 1.9 mg h^−1^ ml_resin_^−1^ in the PCC step). A further increase of the inlet concentration of 45% to 3.2 mg ml^−1^ (Experiment 3) increased the overall productivity by 32% to 7.56 mg h^−1^ (2.52 mg h^−1^ ml_resin_^−1^ in the PCC step). This can be explained by the interplay of several effects. A main effect is that decreasing the inlet stream concentration enables longer cycles of the flow‐through chromatography step, which increases the volumetric throughput hence increasing the loading flow rate of the PCC step (see Table 1) compensating the lower product concentration. While the loading flow rate of the PCC step during Experiment 1 (1 mg ml^−1^) was 0.7 ml min^−1^ the flow rate was 0.5 ml min^−1^ in Experiment 2 (2.2 mg ml^−1^) and 0.45 ml min^−1^ in Experiment 3 (3.2 mg ml^−1^) (see Table 1).

Notably, the relationship between feed concentration and throughput of the flow‐through step is also not linear. First, the time needed to regenerate the column (washing and equilibration) can be considered to be independent of the loading time and therefore leads to a nonlinear relationship. Second, similar to the binding capacity of Capto™ MMC the binding capacity of the flow‐through step on Capto^TM^ Q does also increase with increasing inlet concentration. The amount of DNA that could be removed per flow‐through cycle increased from 272 µg ml_resin_^−1^ to 330 µg ml_resin_^−1^ to 431 µg ml_resin_^−1^, thus leading to higher productivity of the flow‐through step and by extension the overall process.

Although higher concentrations in the starting material generally increase overall productivity, precise prediction of the extent of this increase is complicated as lower concentrations allow higher flowrates, but also change the binding capacities of the resins. In particular, the binding capacities seem to have a tremendous effect on the productivity, which also has been recently described for continuous 2‐column processes in general (Sun et al., 2021). Further in‐depth analysis of the interplay of different process parameters and the influence of the feed stream concentration needs to be conducted, and optimal ratios of resin volumes of the flow‐through step to the PCC step in bind and elute need to be found. This might challenge upscaling as a rational selection of the required column volumes is currently not possible. The dependence of the performance on the feed concentration might limit a designed process to a certain concentration range which negatively affects the flexibility of the set‐up. Recent developments in mechanistic modeling and the construction of so‐called digital twins, might be a powerful tool for decision making, but needs to be extended to integrated processes.

### 3.3 Integrated process product quality

**Figure 5:**
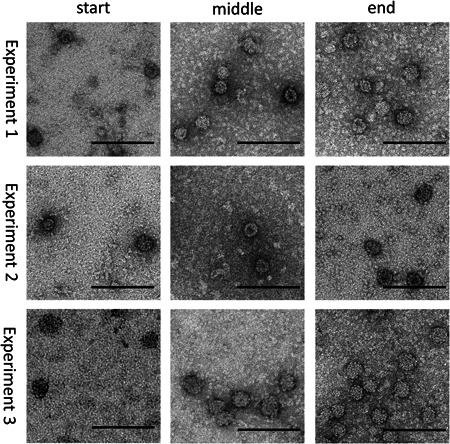
Transmission electron microscopy images of virus‐like particles obtained at different times during continuous processing. Scale bar represents 200 nm.

**Figure 6:**
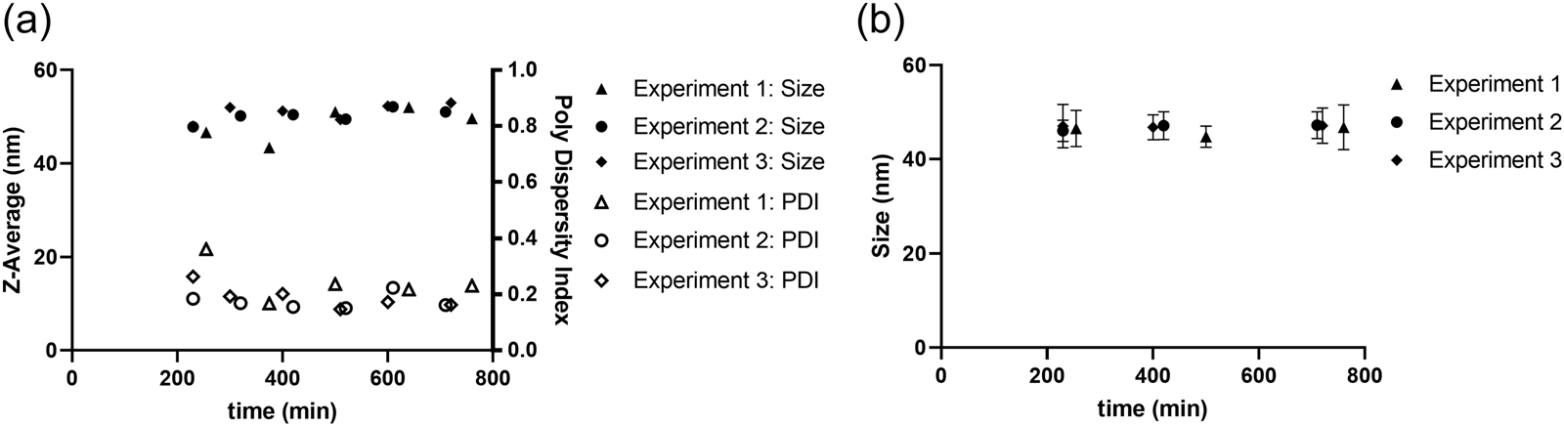
Variation of virus‐like particles diameter (measured by dynamic light scattering [A] and transmission electron microscopy [B]) with process time. Runs were conducted with initial protein concentrations of 1 mg ml^−1^ (Experiment 1), 2.2 mg ml^−1^ (Experiment 2), and 3.2 mg ml^−1^ (Experiment 3).

TEM images of VLPs collected at different time points at the outlet of the process are presented in Figure 5 and size analysis by counting pixels from TEM and analysis by DLS are presented in Figure 6. The optimized process produced highly uniform VLPs with a mean diameter of the samples, measured by counting pixels, ranging between 44.8 ± 2.3 and 47.2 ± 2.9 nm for all three different initial protein concentrations over the entire experiments; no trend in the size could be observed. There are no aggregates visible in the samples, however, all images show nonassembled capsomeres similar to batch processing in our recent publication and other published work (Chuan et al., 2010; Gerstweiler et al., 2021c). Also, there is no apparent difference in the quality of VLPs produced at the beginning of the process compared to VLPs produced toward the end of the process (Figure 5).

Examining the assembly products using DLS shows a similar result. The intensity weighted mean hydrodynamic size (Z‐average) for samples taken during the experiments is shown in Figure 6. All three experiments show nearly steady Z‐averages of the VLPs between 46.6 and 53.0 nm during the entire process (except the second sample of the first experiment which shows a Z‐average of only 43.4 nm, which we cannot explain), slightly higher than by counting pixels. This difference is likely a consequence of parameters used in DLS analysis and/or the effect of preparation of samples for TEM. It is well known that DLS is measuring the average hydrodynamic diameter which is dependent on ionic strength, temperature, and buffer composition.

There is a slight upward trend in the Z‐average diameters as the first samples of each experiment have Z‐averages of 46.6, 47.8, and 48.0 nm, respectively. While samples taken later in the process show Z‐averages slightly above 50 nm. The upward trend in DLS size, that cannot be observed by counting pixels, might be caused by the higher proportion of unassembled capsomeres compared to assembled VLPs in the early process, caused by low VP1‐J8 concentrations of 0.26, 0.29, and 0.37 mg ml^−1^. It was shown in literature, that during assembly of VP1 around 0.02 mg ml^−1^ capsomeres remain unassembled and therefore a lower overall VP1‐J8 capsomere concentration negatively influences the ratio of unassembled capsomeres to assembled VLPs (Ding et al., 2010). The PDI remains relatively stable during the experiments but is slightly higher at the beginning of each experiment. This again can be explained by the low concentration of the sample and the consequential higher unassembled capsomere content. The average PDI of Experiments 1–3 are 0.243 ± 0.064, 0.174 ± 0.025, and 0.190 ± 0.037, respectively. Which are within acceptable limits, although Experiment 1 is slightly elevated (Danaei et al., 2018). The slightly higher PDI of Experiment 1 might be also explained by the lower concentration, but it can also be a random error and requires further investigation.

Both measured sizes of approximately 47 nm by counting pixels on TEM and a Z‐average slightly above 50 nm matches well with reported sizes in literature of a murine poliomavirus VLPs, that reports a Z‐average of 52.0 nm and TEM sizes up to 48 nm (Chuan et al., 2008).

The instruments and process conditions used for assembly of VLPs could be improved further to achieve enhanced VLP yield and by reducing the level of nonassembled capsomeres. Knowing the complexity of in vitro VLP assembly, finding optimal assembly conditions is challenging and requires the consideration of suitable pH, ionic strength/salt type, and temperature conditions (Le & Müller, 2021). As the capsomeres have the same protein composition as the VLPs, we do not anticipate that these represent a product contaminant per se. Our previous work has demonstrated that capsomeres invoke a similar quality of immune response to that of VLPs, albeit at a lower level in the absence of adjuvant (Wibowo et al., 2013).

**Figure 7:**
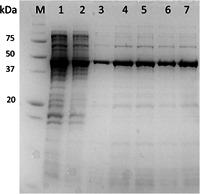
SDS‐PAGE analysis of the process. M: protein marker; (1) Crude lysate from Experiment 2; (2) After flow‐through chromatography purification on Capto™ Q from Experiment 2; (3) After periodic counter‐current chromatography (PCC) step on Capto™ MMC from Experiment 2, start of the process; (4) After PCC step on Capto™ MMC from Experiment 2, middle of the process; (5) After PCC step on Capto™ MMC from Experiment 2, end of the process; (6) After PCC step on Capto™ MMC from Experiment 1, end of the process; (7) After PCC on Capto™ MMC from Experiment 3, end of the process.

SDS‐PAGE analysis (Figure 7) shows a good purity with some contaminations of VP1‐J8 after purification with Capto™ Q and Capto™ MMC. Samples taken at different times during Experiment 2 (Figure 7, lanes 3–5) show no difference in the purity, however, sample concentration at the beginning of the process is too low, to show impurities. Also, comparing purities of the three experiments (Figure 7, lanes 5–7), each taken toward the end of the process, reveals no apparent difference in the purity. In one of our recent publications, we could show, that the low molecular weight impurities are mainly VP1‐J8 truncation products, that were hard to remove, even with further purification. The overall purity equals to the purity obtained in batch processes (Gerstweiler et al., 2021c).

**Figure 8:**
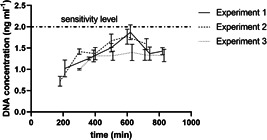
Variation in DNA concentration during virus‐like particles processing. Runs were conducted with initial protein concentrations of 1 mg ml^−1^ (Experiment 1), 2.2 mg ml^−1^ (Experiment 2), and 3.2 mg ml^−1^ (Experiment 3).

Similarly, the DNA concentration of all samples was measured to be between 0.7 and 1.7 ng ml^−1^ at VP1‐J8 concentrations between 0.43 and 0.7 mg ml^−1^ and remains stable during the experiments (Figure 8). Given the fact that the sensitivity level of the assay is 2 ng ml^−1^ it can be assumed the samples are effectively DNA free. As vaccines are given at very low dose of for example only 20 µg protein per dose, DNA levels are expected to be at least 3 magnitudes lower than permitted levels (Australian Government, 2022; WHO, 2010).

Based on these outcomes, it is evident that the described process can produce constantly high‐quality VLP vaccines continuously over the examined duration of at least 10 h. The product quality is in this study independent of the feed concentration, but the overall productivity increases with increased feed concentrations.

Integrating a flow‐through chromatography step with a subsequent PCC bind‐elute process using salt tolerant mixed‐mode chromatography resins (Capto™ MMC) is a powerful approach for continuous biomanufacturing of VLP vaccines. The biggest advantage is that it enables a streamlined process without any intermediate buffer adjustment between the two unit‐operations and therefore theoretically enables a seamless integration without any holding vessel. Like in the previously developed batch process, this set‐up also allows impurities to be reduced to a level that enables UV‐based control of the column loading and a buffer exchange during elution that enables the removal of stabilizing DTT without a dedicated buffer exchange step (Gerstweiler et al., 2021c).

The process fulfils several proposed benefits of continuous processing. Once set‐up the entire process runs automatically and no user interference is required. As sown in Figure 2, applying continuous PCC instead of batch‐wise bind‐and‐elute, allows for much higher column loading, without product loss, and therefore solves the trade‐off between column utilization and productivity. Furthermore, this leads to smaller required columns and a decrease in buffer consumption compared to batch processing. The overall footprint is only 4 m bench space as only minimal hold‐up vessels are needed between the unit operations. The footprint would also not increase much if the process is scaled‐up by increasing resin volumes. Continuous processing also decreases the mean residence time of the product as holding times between unit operations are minimal and do not vary between batch‐to‐batch. This can lead to a better product quality and less quality variations compared to batch processing. It is well known that long processing and hold‐up times can lead to product loss caused by aggregation and proteolytic degradation and our previous study indicates that a quick processing is beneficial for the quality of VP1 (FDA, 2019; Gerstweiler et al., 2021c; Joshi et al., 2014; Ryan & Henehan, 2013). Furthermore, a higher product output can be achieved by solely running the process for a longer time.

We believe that this set‐up can be used as a template for continuous processing of many biologics other than VLPs and viral capsomeres. Although the performance of this process using other entities than VP1‐J8 has not been tested the combination of a flow‐through step with a bind and elute PCC is not product specific. There is also room for adapting the process by changing resins and the substitution of the assembly unit operation with a final flow‐through polishing. This will likely allow the processing of a wide variety of biologics.

## 4 CONCLUSION

Here, we report for the first time an integrated and continuous downstream process for the production of VLP vaccines. Coupling a flow‐through step with a bind and elute PCC process allows for a streamlined process without buffer adjustment between the unit operations. Buffer exchange during elution prepared for a direct VLP assembly by adding calcium ions and pH adjustment thereafter. The process showed a robust behavior toward different inlet concentrations and was capable of producing VLPs of constant high product quality continuously with an outstanding product recovery of 86% and an overall output of up to 7.56 mg h^−1^ with only 8 ml of chromatography resin used in the entire process, and a productivity of the PCC process of 2.52 mg h^−1^ ml_resin_^−1^. Furthermore, the entire process can be assembled on 4 m of lab space, which will only minimally increase if larger column volumes are used, as only minimal holding vessels are required. This clearly shows the dramatically reduced footprint that continuous processing can achieve.

Finding the optimal design space for the highest productivity is challenging as different inlet concentrations lead to changes in the binding capacity and flowrates; and further research needs to be done on how to optimize the described process. As the combination of a flow‐through step with a subsequent PCC step using mixed‐mode resins allows for a wide design space and is not product specific, we believe that the described process can be easily adapted as a template for the development of continuous processing of VLPs and other biopharmaceuticals.

## AUTHOR CONTRIBUTIONS

Lukas Gerstweiler conceived the original research idea, designed and performed the study, wrote the manuscript, and carried out experimental work. Jagan Billakanti contributed to conceptualization, experimental design and analysis, and writing. Jingxiu Bi supervised the project and assisted with conceptualization and writing. Anton P. J. Middelberg supervised the project and contributed to conceptualization, experimental design and analysis, and writing. All authors reviewed and approved the manuscript.

## CONFLICTS OF INTEREST

The authors declare no conflicts of interest.

## Figures and Tables

**Figure A1:**
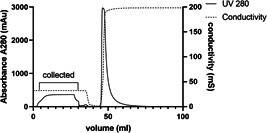
Chromatogram of the flow‐through step on Capto™ Q, showing UV 280 nm absorbance and conductivity. The product flow‐through is collected as highlighted.

**Figure A2:**
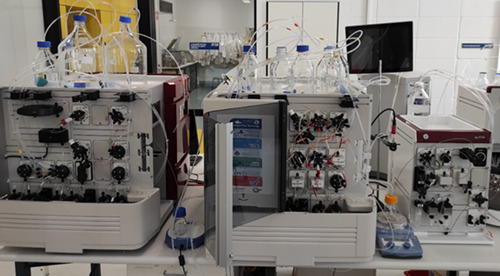
Set‐up of the continuous process consisting of a flow‐through step (left), a periodic counter‐current chromatography step (middle) and an in‐line assembly step (right). The unit operations were coupled using 50 ml stirred tank vessels, and 3 Äkta systems were used to implement the process. A schematic process diagram can be found in Figure 1.

## Data Availability

The data that support the findings of this study are available from the corresponding author upon reasonable request.

## 1

Figure A1, Figure A2.
